# Five Years Pathological Evaluation of Corneal Regrafts: A Study from Southern Iran

**DOI:** 10.1155/2020/2546923

**Published:** 2020-11-04

**Authors:** Masoomeh Eghtedari, Mahmood Kamalzadeh, Masoud Yasemi, Hossein Movahedan, Mohammad Javad Ashraf

**Affiliations:** ^1^Poostchi Ophthalmology Research Center, Department of Ophthalmology, School of Medicine, Shiraz University of Medical Sciences, Shiraz, Iran; ^2^Associate Professor, Department of Pathology, Shiraz University of Medical Sciences, Shiraz, Iran

## Abstract

**Purpose:**

Corneal regrafts sometimes needed to restore the transparency after graft failure. The aim of the study is five years epidemiologic and histopathological evaluation of corneal regrafts.

**Methods:**

In this cross-sectional study, all corneal regrafts during 5 years (2012–2016) were assessed in the Khalili Ophthalmology Center at Shiraz city. Demographic data including age, area of residence, primary disease, type of graft, cause of regraft, interval between primary and subsequent grafts (IPSG), associated eye diseases or surgeries, and systemic diseases were recorded. Also, microscopic findings of corneas were reviewed.

**Results:**

Among a total of 1190 corneal grafts, 76 of them (6.38%) were regrafts. The most common type of grafting was penetrating keratoplasty (PK). The shortest IPSG was observed in fungal keratitis. Main causes of graft failure were endothelial dysfunction, infection, immunologic rejection, technical problems, and recurrence of primary disease, respectively. The most common histopathological finding in failed grafts was severe endothelial cell loss (89.8%). Also, more than half and one-third of cases had Descemet membrane changes and stromal ingrowth, respectively.

**Conclusion:**

Endothelial cell loss was the major cause of failure in our study. Also, recurrence rate in infective cases, especially fungal keratitis, was very high. Considerable presence of histopathological changes such as doubling of Descemet membrane and retrocorneal fibrous ingrowth need further investigations. Perhaps, modification in techniques of corneal grafting and assessment of donor tissue and recipient bed along with any need for longer medical treatment are the basis for future studies in order to increase graft survival.

## 1. Introduction

Corneal transplant survival depends on many factors such as the quality of the donor tissue, technique of the surgery, postoperative care, and rapid controlling of the consequences occurring afterward such as rejection or infection [1–3]. Postoperative consequences of the corneal graft surgery include wound leak, trapping of the corneal inside the wound, glaucoma, endophthalmitis, insufficiency or impaired function of corneal endothelial cells, persistent defect of epithelium, recurrence of primary disease, and endothelial or stromal rejection. Complications of graft sutures include tight fastening or loosening of the stitches, tearing or infectious abscess, noninfectious infiltration of the stitches, conjunctivitis, and vascularization along the incision [2, 4–6].

Bacterial, fungal, and viral infections are among other serious complications after corneal transplantation [4, 5]. All mentioned consequences, if not treated on time, along with other factors such as trauma, coexisting eye problems such as allergic and immunologic disorders, ocular surface diseases, eyelid and eyelash disorders, systemic diseases with ocular manifestations such as autoimmune diseases, rheumatic diseases, hypothyroidism, and previous intraocular surgeries can threaten the grafted cornea and results in unfavorable vision or pain and inconvenience. Moreover, vascularization, pannus formation, improper fibrous, or fibrovascular ingrowth behind grafted cornea would threaten the outcome and may lead to graft dysfunction [5, 7, 8].

The purpose of this study is to evaluate the epidemiologic and histopathological findings of corneal regrafting conducted during 5 years (2012–2016) at Khalili Hospital, Shiraz, as the major referral center in southern Iran in order to find the main risk factors of corneal regraft and highlight the need for better understanding of pathophysiology of graft failures based on histologic findings.

## 2. Materials and Methods

In the present cross-sectional study, epidemiological and histopathological characteristics of all corneal regraft cases at Khalili Hospital, Shiraz, Iran, were evaluated during a 5-year period (2012–2016). The research project has been approved by Ethics Committee of Shiraz University of Medical Sciences with number 4982–94.

In the first step, all files of corneal grafting conducted in the abovementioned years were assessed. The number and type of the primary corneal grafts were separately extracted, and their records and pathology reports were studied. Seventy-six cases of corneal regraft surgeries were found, and data were retrieved from their files. Patients' records including file number, pathology number, age, gender, area of residence (province and urban versus rural), primary diagnosis, type of the regraft, interval between surgeries (first and second graft survival), the reason for regraft, history of coexisting eye disease, and presence of systemic disease were recorded in the designated forms. Then, hematoxylin and eosin- (H&E-) stained tissue slides were reviewed by an expert ocular pathologist using an Olympus BX40 microscope. Histological findings were evaluated, including changes in epithelium, subepithelium, acute or chronic inflammation in the stroma, keratocyte changes, presence of scar, deposits, necrosis, Descemet membrane changes, endothelial cell loss, and ingrowth of any fibro or fibrovascular tissue behind the cornea.

### 2.1. Statistical Analysis

For better analysis, the patients were divided to nine groups with 10 years intervals (from 0–10 to 80–90 years). Finally, the data were analyzed with Statistical Package for Social Science (SPSS version 18) software. Data are presented as mean ± standard deviation and percentages. The chi-square test was used for comparison of data. *P* value of less than 0.05 was considered to be statistically significant.

## 3. Results

During 5 years (2012–2016), 1190 cases of corneal transplants were conducted at Khalili Hospital, Shiraz. Among them, 800 (67.2%) were penetrating keratoplasty (PK), 250 (21%) were deep anterior lamellar keratoplasty (DALK), and 140 (11.7%) were Descemet striping endothelial keratoplasty (DSAEK). Hence, among 800 cases of PK during a 5-year period, 66 cases (8.25%) ended up with regrafting, and among 250 cases of DALK and 140 cases of DSAEK, only 3 (1.2%) and 7 cases (5%) resulted in regrafting, respectively.

The overall rate of corneal regraft was 6.38% (76 cases) during five years, and among them, 50 cases (65.8%) were men. There was a statistically significant difference (almost twice more in men) between male and female (*P*  value = 0.01).

Average age at the time of corneal regraft surgery was 55 years with standard deviation (SD) of 6. Also, the highest and lowest age was 84 years and 2 years, respectively.

**Figure 1:**
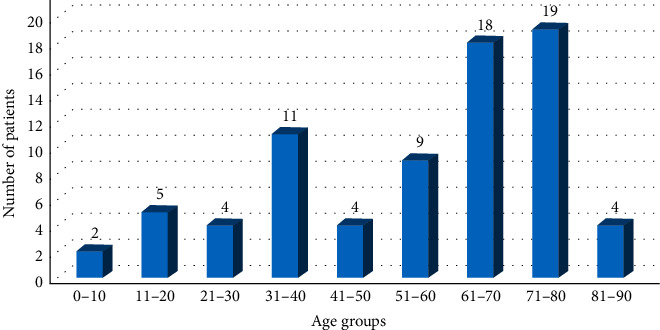
Patient's numbers with regrafted cornea in different age groups.

The number of patients with corneal regraft in different age groups is shown in Figure 1.

Based on it, patients in the age groups of 70–80 and 60–70 have been the remarkably highest rate of corneal regraft, respectively. However, the association between increasing age and rate of corneal regraft was not statistically significant (*P*  value = 0.12).

Regarding the residential area, Fars Province had the largest number of referrals (44 out of 76), followed by Bushehr Province (8 cases), Hormozgan (7 cases), Kohgiluyeh (6 cases), and Khuzestan (5 cases). The rest were from other neighbor provinces.

The most prevalent cause of primary corneal transplantation in patients who had later undergone corneal regraft surgery was pseudophakic or aphakic bullous keratopathy (30.3%) that followed by herpetic (14.5%), fungal (11.8%), and bacterial (9.2%) corneal infections. Infective causes all together (35.5%) were the main causes for primary transplants in regraft cases (27 cases). Among all graft failures resulted in second surgery, recurrence of primary disease was the cause of failure in 14 cases (7 herpetic infection, 4 fungal ulcers, and 2 bacterial keratitis recurred 6–8 months after primary surgery and one gelatinous dystrophy). Prevalence of different causes of corneal regrafts is shown in the Table 1.

Secondary procedure in the vast majority of cases (74 out of 76 regrafts) was PK; two cases underwent DSAEK, both carried out on previous failed PKs. In two DSAEK cases, regraft was needed due to the technical problem of primary procedure; one was performed 1.5 months and the other one 24 months after primary operation.

The average time interval between first and second procedures was 47 months. The shortest interval was 1.5 months (PK was carried out because of failed DSAEK). The longest graft survival belongs to PBK cases. Also, 13% of regrafts cases had glaucoma.

Underlying systemic diseases (9.2% hypertension, 6.5% diabetes mellitus, 3.9% thyroid disease, and 2.6% rheumatologic disorder) were found in 22.2% of the patients.

In regards to microscopic evaluation, epithelial changes were observed in 86.8% of cases (including defect (33%), irregularity (55.3%), edema and bulla (39.4%), thinning (10.5%), thickening (2.6%), and keratinization (1.3%)). There is a statistically significant relationship between epithelial abnormalities and graft failure resulting in corneal regrafts (*P*  value = 0.005). Subepithelial pannus was observed in 25% of regraft cases. This finding was particularly common in the rejection cases (54.5%). Bowman's layer abnormalities were observed in 85.5% of regraft cases (*P*  value = 0.04). The most common abnormality of Bowman's layer was disruption (63.2%). There is a significant association between vascularization and graft failures, especially in cases of rejection and herpetic ulcers (*P*  value = 0.005).

Retrocorneal fibrous ingrowth was observed in 23 cases (30.3%). There was a statistically significant association between regraft cases and ingrowth. No statistically significant association was found between systemic diseases and presence of fibrous ingrowth.

Forty cases showed Descemet membrane changes (52.6%), and the most common pathology was “Excretions” (13.2%). Prevalence of Descemet membrane changes in the different causes of corneal regrafts are given in Table 2.

Also, 71 out of 76 regrafts (93.4%) showed endothelial disorders. Details of histopathological findings of corneal regrafts cases are given in Table 3.

**Figure 2:**
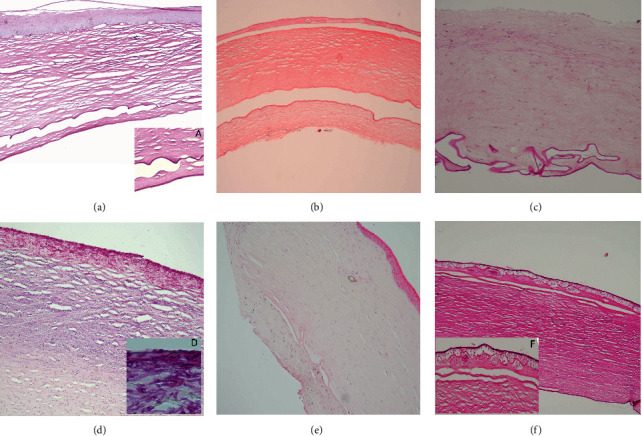
Pathological findings in the regrafted corneas with staining of periodic acid-Schiff (PAS). (a) Descemet membrane doubling and fibrous ingrowth (magnification; (a) ×40 and (b) ×400). (c) Descemet membrane misplacement (×40). (d) Descemet membrane excretions (×200). (e) Fungal keratitis ((e) ×40 and (f) ×400). (g) Retrocorneal fibrous ingrowth (×100). (h) subepithelial pannus ((h) ×40 and (i) ×100).

Pathological findings of regrafted corneas with periodic acid-Schiff (PAS) staining are shown in Figure 2.

## 4. Discussion

The main goal of corneal transplant is to restore vision. Because of immune privilege of the cornea, the initial success of corneal graft is often high and close to 90%; however, the graft survival rate is still affected by serious causes [9, 10].

Based on our results, the rate of regraft in all transplanted corneas during 5 years was 6.38% and is similar to the failure rate (5–30%) in other studies [2, 7, 11, 12]. Also, DALK was the most successful type of grafting with the regraft rate of 1.2% followed by DSAEK (5%) and PK (8.25%). Most probably, the nature of primary disease (keratoconus in most of our DALK cases), younger age at the time of surgery, and retaining recipients' endothelium had a significant impact on outcome of surgery. Among 7 cases where the primary procedure was DSAEK, endothelial dysfunction was the reason for regrafting in four of them. Considering the time interval between surgeries (1.5–12 months), the failure was most probably caused by technical problems [13]. Considering less familiarity of our cornea surgeons with DSAEK technique at the beginning years of its introduction, the success rate was still very acceptable.

In the present study, endothelial dysfunction of grafted cornea was the main cause of corneal regraft, in accordance with most similar studies [4, 5, 12]; however, infectious causes had a more significant role compared with other studies. It can be due to climacteric environmental differences or social characteristics of most of our patients who are not able to follow the recommended care plans.

Similarly, the number of regrafting cases in men was approximately twice more than women. This gender difference has not been reported in other studies and can be attributed to the cultural-social difference in our society where men usually pay less attention to their healthcare compared to women. In diabetic patients, it has been shown previously that male patients have less compliance for treatment [14]. Nevertheless, the role of hormonal and physiologic factors cannot be ignored and should be further studied.

Based on our study, primary disease of cornea leading to graft was proposed to be the most important indicator of graft survival. Infection (bacterial, herpetic, or fungal) was the major cause for second and even third transplant surgery. Thirteen cases had recurrence of infection (herpetic, fungal, or bacterial). This fact highlights the risk of reinfection in transplanted corneas due to ulcer and emphasizes on the importance of continuing antibiotics or antiviral treatments after surgery. Also, meticulous surgical technique regarding the trephination size and location will help to remove the main focus of infection while keeping a safe margin to the limbal area, if possible. However, based on a recent cohort study, despite the various changes in the methods of corneal transplant surgery in the past 30 years, these methods have no considerable impact on graft survival compared with other factors [15].

The quality of donor cornea may also be an issue as well. At this center, the higher quality buttons usually are preserved for optical keratoplasties, while moderate quality corneas are acceptable for emergency cases and tectonic purposes. Reasonably, lower quality donor corneas fail more easily than higher quality ones, still not a direct explanation for reinfection. Glaucoma could be another reason for endothelial cell damage and found in a significant number of our cases, which is similar to other studies [5].

In histopathological investigation of the regrafting cases, the epithelial disorders were widely observed, which is in accordance with previous studies [16]. Also, the existence of pannus was observed in 25% of regrafts, which may have contribution to graft failure, and is in accordance with previous studies [4]. Observing the high number of subepithelial pannus in rejection cases is another interesting finding in our study.

Alteration of Bowman's layer was observed in a remarkable number of cases. This finding has been reported before [16]. Vascularization of the corneal stroma in the present study was associated with the graft failure and observed in the majority of cases (46%) especially in the immunologic rejections and herpetic keratitis. This finding was observed in previous studies too [7, 17] and considered as a risk factor for graft failure in some of them.

Another interesting finding in this study was Descemet membrane changes including doubling and excretions.

Large number of cases especially those with endothelial abnormalities and immunologic rejection showed these changes (62% and 45% respectively). Apparently, Descemet membrane abnormalities are considered as the sign of endothelial cell dysfunction [12, 18]. Further investigation is required in terms of understanding pathophysiology of these abnormalities.

The growth of fibrous or fibrovascular tissue behind the grafted cornea was observed in one-third of our patients and especially was evident in cases of endothelial dysfunction with thickened corneas. Based on literature review [19–21], the pathology could be due to the discrepancy in stromal thickness between donor and recipient corneas at the appositional circle, which may result in excessive growth of host stromal tissue under donor Descemet membrane. Also, doubling of Descemet membrane may be caused by migration of the host endothelium behind that tissue and production of new Descemet membrane (which could be abnormally thick or thin) [22].

Based on different studies, high resolution corneal imaging (such as optical coherence tomography [23] and confocal microscopy [20]) is a practical technique for evaluating the posterior graft-host junctions' appositions in the cases of penetrating keratoplasty.

Almost all cases of regrafts in our center (93.4%) showed endothelial cell loss or abnormalities; therefore, this finding could be considered as an inevitable outcome in majority of corneal graft failures of any cause. This fact has been shown in previous studies as well [4].

Also, we found a significant association between the presence of retrocorneal fibrous ingrowth with other ocular disorder such as ocular surface disease (in 42%) or previous ocular surgery (in 46%). It emphasizes on the need for treatment of any ocular disease before and after keratoplasty. Like the present study, in many studies, glaucoma has been considered as a major risk factor for graft failure [4, 5, 24, 25]. In our study, diabetes was found in 6.5% of the patients; other studies considered it as a major risk factor for graft failure [16].

Overall, based on the present study, we can point out some facts: infection and inflammation should be controlled more vigorously after transplants; patients should be warned about complications and the need for regular follow-ups even years after primary graft, and finally, surgical techniques may need to be revised.

## Figures and Tables

**Table 1:** Prevalence of different causes of corneal regrafts.

Causes of regraft	Number	Percentage (%)
Endothelial dysfunction	42	55.3
Infectious keratitis	20	26.3
Rejection	11	14.5
Technical	2	2.6
Corneal dystrophy	1	1.3
Total	76	100

**Table 2:** Prevalence of Descemet membrane changes in the different causes of corneal regrafts.

	Rupture	Descemetocele	Doubling	Excretion	Thickening	Descemet change (%)
Bacterial ulcer	1	2	0	0	0	8.1
Fungal ulcer	3	0	0	0	0	8.1
Herpetic ulcer	1	0	0	1	0	5.4
Rejection	0	0	2	3	0	13.5
Endothelial dysfunction	1	0	10	9	4	64.9

**Table 3:** Histopathological findings of corneal regrafts.

Corneal layers	Pathological findings	Number	Percentage
Epithelium	Defect	25	33
	Irregularity	42	55.3
	Edema and bulla	30	39.4
	Thickening	2	2.6
	Thinning	8	10.5
	Keratinization	1	1.3
	Total	108	86.8

Subepithelium	Fibrous pannus	14	18.4
	Fibrovascular pannus	5	6.6
	Total	19	25

Bowman	Disruption	48	63.2
	Irregularity	9	11.8
	Absence	5	6.6
	Thickening	2	2.6
	Band shape keratopathy	1	1.3
	Total	65	85.5

Stroma	Vascularization	35	46
	Increasing Keratocyte	2	2.4
	Decreasing Keratocyte	26	30.6
	Scar	22	28.9
	Ingrowth	23	30.3

Descemet	Doubling	9	12.7
	Thickening	4	5.6
	Excretions	10	14.1
	Doubling and excretions	3	4.2
	Misplacement	1	1.4
	Descemetocele	2	2.8
	Total	35	52.6

Endothelium	Mild cell loss	3	3.9
	Severe cell loss	68	89.8
	Total	71	93.4

## Data Availability

The data used to support this study are available from the corresponding author upon request.
